# The non-linear relationship between triglyceride-glucose index and risk of chronic kidney disease in hypertensive patients with abnormal glucose metabolism: A cohort study

**DOI:** 10.3389/fmed.2022.1018083

**Published:** 2022-09-20

**Authors:** Qing Zhu, Yuan Chen, Xintian Cai, Li Cai, Jing Hong, Qin Luo, Yingli Ren, Yanying Guo, Nanfang Li

**Affiliations:** ^1^Graduate School, Xinjiang Medical University, Ürümqi, China; ^2^Hypertension Center of People’s Hospital of Xinjiang Uygur Autonomous Region, Ürümqi, China; ^3^Department of Endocrinology and Metabolic Diseases of People’s Hospital of Xinjiang Uygur Autonomous Region, Ürümqi, China

**Keywords:** triglyceride-glucose index, diabetes, chronic kidney disease, hypertension, insulin resistance

## Abstract

**Background:**

Triglyceride–glucose (TyG) index has been reported to be associated with cardiovascular disease (CVD). However, few studies have focused on TyG index and the risk of chronic kidney disease (CKD). Thus, this study aims to explore the relationship between TyG index and CKD.

**Methods:**

A total of 2,033 participants with hypertension between January 2012 and May 2019 were included in the longitudinal observational study. All patients are grouped according to the TyG index quartile. CKD was defined as estimated glomerular filtration rate (eGFR) < 60 ml/min per 1.73 m^2^ and/or positive proteinuria. Multivariate Cox proportional hazards models were used to investigate the relationship between TyG index and CKD.

**Results:**

During a median follow-up of 31 months, 302 participants developed CKD, with a mean age of 55.5 years and median TyG of 8.94. Compared with those in the lowest quartile of TyG index, participants in the highest quartile of TyG index exhibited 1.63-fold higher hazard ratio (95% CI: 1.14–2.33, *P* = 0.007) for presence of CKD. And restricted cubic spline analysis showed the relationship between TyG index and CKD is non-linear (P non-linearity = 0.021). The hazard ratio for CKD first fell and after rising until around 8.94 of TyG index and started to increase rapidly afterward (P for TyG < 0.001).

**Conclusion:**

Higher TyG index is associated with the increased risk for CKD. Early intervention of metabolic factors may prevent the occurrence of CKD, thereby reducing the incidence of CVD and premature death.

## Background

Chronic kidney disease (CKD) is an important and common condition globally and was association with cardiovascular events and premature death (1, 2). Both early prevention and treatment of CKD are critical.

CKD occurs commonly in the general adult population, and is closely related to hypertension and diabetes mellitus (DM) (3). Previous studies have shown that patients with hypertension and DM have a higher incidence of renal dysfunction than patients with either hypertension or DM alone (4, 5), suggesting higher risk for CKD in patients with coexistent hypertension and DM. Hypertension and DM are both heterogeneous polygenic genetic diseases, and their common familial aggregation and sequential coexistence are very high (6, 7), indicating that they may have a common pathogenesis. The main and core comorbidity mechanisms are insulin resistance (IR) (8, 9). IR could lead to vascular damage and is associated with several groups of abnormal syndromes that include obesity, DM, cardiovascular disease (CVD), CKD and other abnormalities (10, 11). Therefore, monitoring IR is of great significance for the prevention and treatment of hypertension, DM, and their complications (12).

Triglyceride-glucose (TyG) index, calculated with fasting plasma glucose and triglycerides, had been shown to be significantly associated with insulin resistance and proposed as reliable surrogate marker of IR (13). Recently, lots of studies have indicated the association of TyG index with myocardial infarction, arterial stiffness, stroke, and nephritic microvascular damage (13–16). However, few studies have focused on the association between TyG index and the risk of CKD, specifically in patients with hypertension and abnormal glucose metabolism, a high-risk group for CKD. Thus, the present study was aimed to investigate the relationship of TyG index at baseline with the risk of CKD based on a retrospective cohort study.

## Materials and methods

### Study population

**FIGURE 1:**
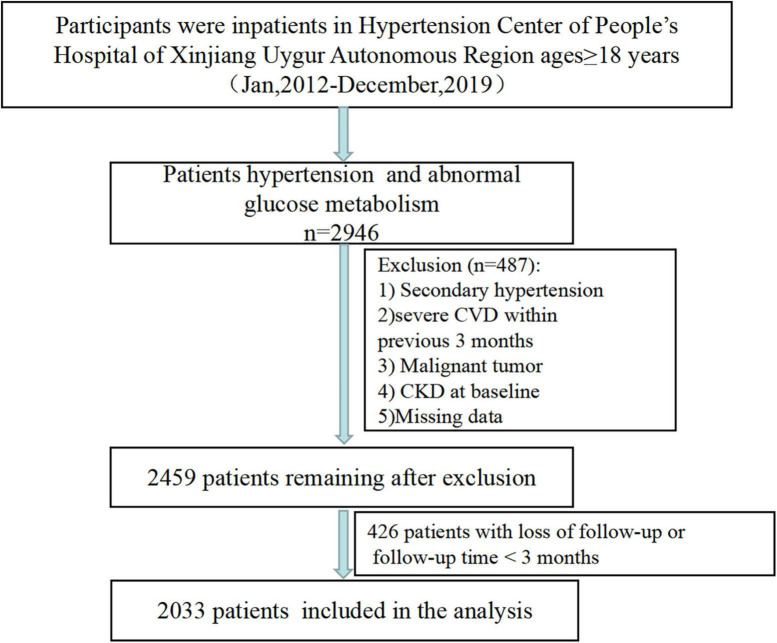
Patient screening flowchart.

Participants were inpatients from Hypertension Center of People’s Hospital of Xinjiang Uygur Autonomous Region in this retrospective study. The study design have been reported elsewhere (17, 18). In brief, a total of 2,946 participants aged ≥ 18 years who diagnosed with hypertension and abnormal glucose metabolism between January 2012 and May 2019, were identified. Individuals diagnosed secondary hypertension, history of CVD within last 3 months (including acute coronary syndrome, coronary revascularization, and coronary bypass operation, heart failure and acute cerebrovascular disease), or malignant tumor, or with CKD at baseline (*n* = 410), or missing baseline data (*n* = 77) were excluded. Finally, we collected the outcome data for 2,459 patients (Figure 1).

### Data definitions

Fasting blood glucose (FBG) and triglyceride (TG) levels were measured by the hexokinase/glucose-6-phosphate dehydrogenase method and the enzymatic colorimetric method, respectively. TyG index was calculated as ln [TG (mg/dL) × FBG (mg/dL)/2]. The units of TG and FBG were converted from mmol/L to mg/dL before calculation (For TG: 1 mmol/l = 88.57 mg/dl; For FBG, 1 mmol/l = 18 mg/dL).

Diagnostic criteria for hypertension and abnormal glucose metabolism [including impaired fasting glucose (IFG), impaired glucose tolerance (IGT), and diagnosed DM] have been described in previous studies (17, 18).

### Follow-up and outcome

Follow-up data were obtained through outpatient or rehospitalization follow-up records, or annual physical examination data. At the same time, for patients with rehospitalization, we also extracted the diagnostic name and diagnostic disease Code (ICD-10) from the patient’s electronic medical record to identify definite CKD to exclude false positive cases caused by transient creatinine elevation or proteinuria of various causes. Retrospective follow-up data were collected until the outcome (new-onset CKD) or until the end of the study (May 31, 2021). Patients were considered lost to follow-up if there were no records of any follow-up visits after discharge and no annual health examination data. In addition, patients who were followed up for less than 3 months after baseline were also considered lost to follow-up. A total of 426 patients were lost to follow-up (Figure 1).

If a participant experienced the outcomes more than once during follow-up, only the first outcome was used for analysis. For those without CKD during follow-up, the data of the last follow-up was included in the analysis (17, 18).

CKD was defined as eGFR < 60 mL/min/1.73 m^2^ and/or proteinuria during follow up. We used the modification of diet in renal disease (MDRD) equation to evaluate eGFR. The qualitative results of urinary protein ≥ 1 + by urine dipstick were considered to be proteinuria.

### Statistical analysis

SPSS (version 23.0) and R (version 4.0.5) software were used to analyze data. Continuous variables were expressed as mean (standard deviation) or median (interquartile range), and categorical variables were presented as frequencies with the percentages. Differences of all variables were compared and tested using the analysis of variance (ANOVA) or non-parametric Kruskal-Wallis *H*-test or a Chi-square test, accordingly. The cumulative incidence of CKD was estimated using the Kaplan–Meier method and differences among groups were evaluated by the log-rank test. Multivariate Cox proportional hazard regressions were constructed to estimate the association of TyG index with CKD by calculating the hazard ratio (HR) and 95% confidence interval (CI). Adjustment of covariates was based on the results of comparison between groups and multi-variable linear regression analysis. Multicollinearity was judged if the tolerance was less than 0.1 and the variance inflation factors (VIF) is greater than 3. Model 1 adjusted for sex, age, smoke, drink, BMI, duration of hypertension, duration of diabetes (known risk factors for CKD); Model 2 adjusted for variables in Model 1 plus SBP, DBP, LDL-C, HDL-C, Cr, BUN, and UA (all non-collinearity variables, and with *P* < 0.05 in baseline and univariable Cox analysis). Model 3 further adjusted for all included factors (in Model 2 plus lipid-lowering drugs, antidiabetic drugs, and antihypertensive drugs). Multivariate linear regression was used to evaluate the association between TyG index with eGFR by calculating the B coefficients and 95% CI.

In addition, restricted cubic spline analysis was performed to explore the dose-response relationship between TyG index with CKD, and eGFR. Two-tailed *p*-value < 0.05 was considered statistically significant.

Additionally, stratification analyses and sensitivity analyses were performed to further determine the relationship by excluding individuals with follow-up time less than 12 months.

## Results

### Baseline characteristics

A total of 2,033 participants were successfully followed up and included in the current study (Figure 1), with complete data of FBG and TG. The mean age was 55.53 ± 11.05 years 56.5% were men, 58.1% had diagnosed DM and mean BP were 148/88 mmHg. Baseline characteristics by quartiles of the TyG index were shown in Table 1. Participants with the highest TyG index tended to be younger, men, more current smokers, and alcohol drinkers, have higher prevalence of diabetes, FBG, HbA1c, TC, TG, LDL-C, and UA levels, have lower HDL-C levels, compared with participants in the lowest quartile group.

**TABLE 1:** Baseline characteristics of participants according to quartiles of TyG index.

	TyG (quartiles)				*P*
	Q1 (7.16–8.57) *n* = 509	Q2 (8.58–8.94) *n* = 508	Q3 (8.95-9.40) *n* = 508	Q4 (9.41–12.29) *n* = 508	
Age (years)	56.43 ± 11.71	56.90 ± 10.81	55.06 ± 11.04	53.72 ± 110.34	<0.001
Male ***n* (%)**	287 (56.4)	256 (50.4)	287 (56.5)	319 (62.8)	0.001
Body mass index (kg/m^2^)	27.44 ± 3.94	27.99 ± 3.81	28.27 ± 3.95	28.52 ± 3.89	<0.001
Smoking yes *n* (%)	131 (25.70)	125 (24.60)	155 (30.50)	181 (35.60)	<0.001
Drinking yes *n* (%)	133 (22.20)	115 (22.60)	146 (28.70)	165 (32.50)	<0.001
Duration of hypertension (years)	6 (2–13)	8 (2–13)	6 (2–11.75)	6.5 (3–12)	0.329
Duration of diabetes (years)	25 (18.80)	120 (22.60)	25 (18.80)	120 (22.60)	<0.001
Abnormal glucose metabolism types *n* (%)					<0.001
Impaired fasting glucose	101 (19.8)	59 (11.6)	47 (9.3)	30 (5.9)	
Impaired glucose tolerance	186 (36.5)	175 (34.4)	159 (31.3)	95 (18.7)	
Diabetes mellitus	222 (43.60)	274 (53.9)	302 (59.4)	383 (75.4)	
Systolic BP (mmHg)	148.04 ± 21.52	146.89 ± 20.51	149.19 ± 21.45	149.90 ± 21.28	0.116
Diastolic BP (mmHg)	86.58 ± 15.53	86.20 ± 14.20	88.94 ± 14.79	89.85 ± 14.92	<0.001
Pulse (bits/min)	80.49 ± 10.57	80.90 ± 10.60	82.74 ± 11.29	83.88 ± 11.10	<0.001
FBG (mmol/L)	4.90 ± 0.95	5.44 ± 1.08	6.24 ± 1.83	8.14 ± 3.02	<0.001
HbA1c (%)	6.41 ± 0.82	6.62 ± 0.99	6.91 ± 1.19	7.73 ± 1.67	<0.001
Cholesterol (mmol/l)	3.93 ± 0.94	4.25 ± 0.93	4.59 ± 0.92	4.95 ± 1.29	<0.001
Triglyceride (mmol/l)	1.03 ± 0.27	1.53 ± 0.31	2.02 ± 0.50	4.03 ± 3.08	<0.001
HDL-C (mmol/l)	1.05 ± 0.29	0.99 ± 0.20	0.96 ± 0.22	0.89 ± 0.20	<0.001
LDL-C **(mmol/l)**	2.40 ± 0.85	2.66 ± 0.84	2.87 ± 0.82	2.56 ± 0.87	<0.001
Serum Cr (μmol/L)	66.28 ± 14.51	65.94 ± 15.63	65.99 ± 16.27	65.95 ± 16.32	0.835
Baseline eGFR (ml/min/1.732)	116.23 ± 27.29	115.98 ± 29.29	118.72 ± 29.65	121.76 ± 33.21	0.014
BUN (mmol/L)	5.06 ± 1.43	5.09 ± 1.44	5.05 ± 1.40	5.22 ± 1.33	0.024
Uric acid (μmol/l)	317.20 ± 78.67	333.16 ± 83.67	332.50 ± 81.10	346.18 ± 94.58	<0.001
Serum potassium (mmol/L)	3.66 ± 0.29	3.66 ± 0.27	3.71 ± 0.27	3.68 ± 0.30	0.060
TyG	8.24 ± 0.25	8.76 ± 0.11	9.15 ± 0.14	9.94 ± 0.51	<0.001
Antihypertensive agents *n* (%)					
ACEI/ARB	282 (55.4)	289 (56.9)	282 (55.5)	322 (63.4)	0.030
CCB	407 (80.0)	430 (84.6)	424 (83.5)	416 (81.9)	0.226
Beta-blocker	109 (21.4)	116 (22.8)	102 (20.1)	116 (22.8)	0.667
Diuretics	210 (41.3)	184 (36.2)	159 (31.3)	163 (32.1)	0.003
Hypoglycemic therapy *n* (%)	193 (37.9)	258 (50.8)	296 (58.3)	391 (77.0)	<0.001
Lipid-lowering therapy *n* (%)	405 (79.6)	423 (83.3)	410 (80.7)	429 (84.4)	0.156
Follow-up time (years)	2.78 (1.54–4.26)	2.50 (1.45–4.17)	2.59 (1.53–4.51)	2.43 (1.43–4.11)	0.737

FBG, fasting blood glucose; LDL-C, low-density lipoprotein cholesterol; HDL-C, high-density lipoprotein cholesterol, Cr, creatinine; BUN, Blood urea nitrogen; ACEI, angiotensin-converting-enzyme inhibitors; ARB, angiotensin receptor blockers; CCB, calcium channel blockers.

In addition, baseline characteristics by CKD and not CKD were shown in Supplementary Table 1.

### Association of triglyceride–glucose index with the risk of chronic kidney disease

**FIGURE 2:**
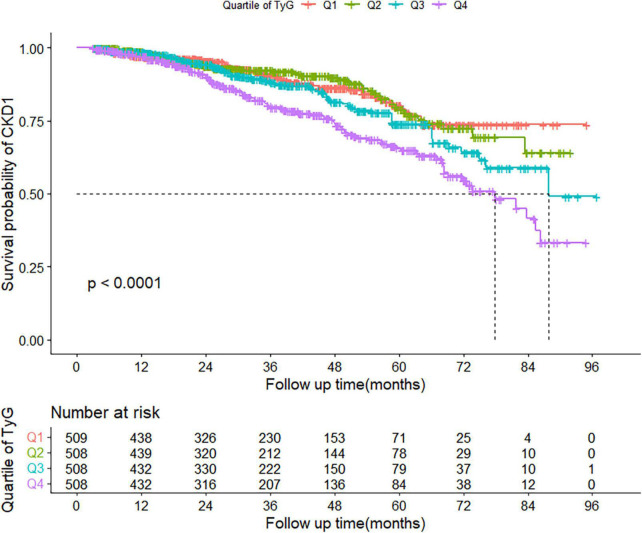
Kaplan–Meier curve of cumulative incidence of CKD based on quartiles of TyG index; *P*-value was generated based on log-rank test. CKD, chronic kidney disease.

During a median follow-up of 31 (interquartile range: 18–51) months, 302 patients subsequently developed CKD. The incidence of CKD increased with increasing TyG index quartiles, from 37.39 in quartile 1 to 68.95 per 1,000 person-years in quartile 4. Participants in quartile 4 of TyG index had a higher risk for CKD, compared to participants in the other groups (log-rank test, *P* < 0.001; Figure 2).

In the collinearity test, FBG, TG, TyG, TC, and LDL-C showed VIF greater than 3; therefore, we excluded FBG, TG and TC in further analysis (shown in Supplementary Table 2).

The association between TyG index and CKD was shown in Table 2. Unadjusted Cox regression showed that TyG index had a positive association with CKD. After adjusting for different confounders (Model 1 and Model 2), this association still exists. Even after all covariates (Model 3), the risk for CKD increased 30% (95% CI, 1.10–1.15, *P* = 0.003) with each one-unit increase of TyG index. Taking the lowest quartile as a reference, the adjusted HRs (95% CIs) in the second, third and highest quartiles of TyG index were 0.97 (0.67–1.43), 1.22 (0.84–1.75), and 1.63 (1.14–2.33), respectively. P for trend was <0.05. The results were similar for its median, tertile, and for each 1 SD increment (Table 2).

**TABLE 2:** Association between TyG and CKD.

	Unadjusted model		Model 1		Model 2		Model 3	
	HR (95% CI)	*P*	HR (95% CI)	*P*	HR (95% CI)	*P*	HR (95% CI)	*P*
TyG	1.46 (1.25–1.70)	<0.001	1.42 (1.21–1.66)	<0.001	1.32 (1.12–1.55)	0.001	1.30 (1.10–1.55)	0.003
TyG per 1 SD	1.30 (1.17–1.44)	<0.001	1.27 (1.14–1.42)	<0.001	1.21 (1.08–1.35)	0.001	1.20 (1.07–1.35)	0.003
lnTyG	1.80 (1.36–2.40)	<0.001	1.69 (1.26–2.26)	<0.001	1.45 (1.08–1.96)	0.014	1.43 (1.04–1.95)	0.026
TyG **Median**								
≤8.94	1		1		1		1	
>**8.94**	1.68 (1.33–2.12)	<0.001	1.64 (1.29–2.08)	<0.001	1.49 (1.17–1.90)	0.001	1.44 (1.11–1.86)	0.005
TyG tertiles								
Tertile 1 (7.16–8.70)	1		1		1		1	
Tertile 2 (8.71–9.22)	1.08 (0.79–1.47)	0.651	1.06 (0.78–1.46)	0.703	1.01 (0.74–1.39)	0.940	0.97 (0.70–1.35)	0.870
Tertile 3 (9.23–12.29)	1.87 (1.41–2.47)	<0.001	1.77 (1.33–2.36)	<0.001	1.59 (1.19–2.12)	0.002	1.51 (1.11–2.06)	0.010
P for trend		<0.001		<0.001		0.001		0.004
TyG quartiles								
Quartile 1 (7.16–8.57)	1		1		1		1	
Quartile 2 (8.58–8.94)	0.98 (0.68–1.43)	0.930	1.01 (0.69–1.47)	0.966	1.00 (0.69–1.47)	0.970	0.97 (0.67–1.43)	0.894
Quartile3 (8.95–9.40)	1.32 (0.93–1.86)	0.118	1.35 (0.95–1.91)	0.091	1.27 (0.89–1.81)	0.182	1.22 (0.84–1.75)	0.294
Quartile 4 (9.41–12.29)	2.03 (1.47–2.79)	<0.001	1.94 (1.40–2.70)	<0.001	1.72 (1.23–2.41)	0.001	1.63 (1.14–2.33)	0.007
P for trend		<0.001		<0.001		<0.001		0.002

Model 1 adjusted sex, age, smoke, drink, BMI, duration of hypertension, duration of diabetes. Model 2 adjusted for variables in Model 1 plus SBP, DBP, LDL-C, HDL-C, Cr, BUN, and UA. Model 3 adjusted for variables in Model 2 plus lipid-lowering drugs, antidiabetic drugs, and antihypertensive drugs.

**FIGURE 3:**
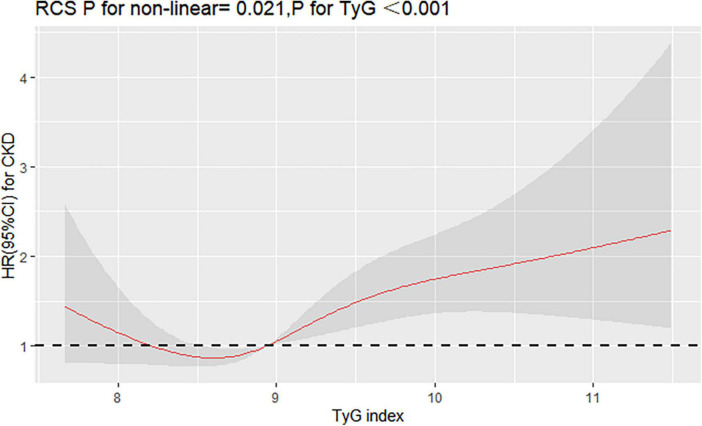
Restricted cubic splines (RCS) for the shape of the association of TyG with CKD. It was used a multivariate Cox regression model of restricted cubic spline with 4 knots (at the 5th, 35th, 65th, and 95th percentiles) of TyG adjusting for potential covariates (including sex, age, smoke, drink, BMI, duration of hypertension, duration of diabetes, SBP, DBP, LDL-C, HDL-C, Cr, BUN, UA, lipid-lowering drugs, antidiabetic drugs, and antihypertensive drugs). The reference point for TyG index was 8.94 (the median of TyG).

**FIGURE 4:**
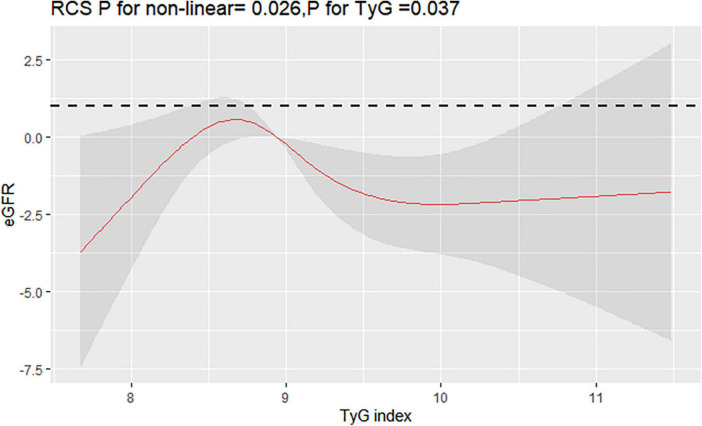
Restricted cubic splines (RCS) for the shape of the association of TyG with eGFR. It was used a multivariate linear regression model of RCS with 4 knots (at the 5th, 35th, 65th, and 95th percentiles) of TyG adjusting for potential covariates (the same as Figure 3). The curve was centered at the median value of 8.94.

Interestingly, restricted cubic spline analysis showed the relationship between TyG index and the risk of CKD is non-linear (P non-linearity = 0.018) (Figure 3). The HR for CKD first fell and after rising until around 8.94 of TyG index (TyG median) and started to increase rapidly afterward (P for TyG < 0.001). When further analyzed the relationship between TyG and eGFR by multivariate linear regression, the results showed that per 1-unit increase of TyG index induced about 2 unit decrease of Egfr (β: –2.12,95% CI:–4.01 to –0.23, *P* = 0.037). And, restricted cubic spline analysis also showed the relationship between TyG index and eGFR is non-linear (*P* = 0.026) (Figure 4).

### Stratification analysis and sensitivity analysis

**FIGURE 5:**
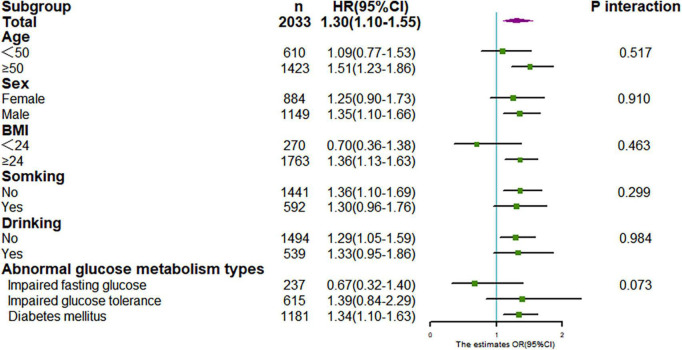
Stratification analysis on association between TyG with CKD. Results were derived from multivariate Cox regression and presented as hazard ratio (adjusting covariates: sex, age, smoke, drink, BMI, duration of hypertension, duration of diabetes, SBP, DBP, LDL-C, HDL-C, Cr, BUN, UA, lipid-lowering drugs, antidiabetic drugs, and antihypertensive drugs).

We performed a series of stratified analysis, and none of the variables significantly modified the association between TyG index and CKD (*P* for all interaction > 0.05) (Figure 5). Also, sensitivity analysis after excluding individuals with follow-up less than 12 months, showed that relationship between TyG index and CKD was still stable (for TyG as continuous variable, adjusted HR:1.30,95% CI:1.10–1.55, *P* = 0.003; for TyG categorical variable, adjusted HR:1.44, 95% CI: 1.11–1.86, *P* = 0.005), as in Table 3.

**TABLE 3:** Sensitivity analyses after excluding patients with follow up less than 12 months.

	Unadjusted model		Adjusted model	
*N* = 1,740	HR (95% CI)	*P*	HR (95% CI)	*P*
TyG	1.55 (1.31–1.82)	<0.001	1.30 (1.10–1.55)	0.003
TyG **median**				
≤8.94	1		1	
>**8.94**	1.79 (1.39–2.31)	<0.001	1.44 (1.11–1.86)	0.005

Adjusted sex, age, smoke, drink, BMI, duration of hypertension, duration of diabetes, SBP, DBP, LDL-C, HDL-C, Cr, BUN, UA, lipid-lowering drugs, antidiabetic drugs, and antihypertensive drugs.

## Discussion

In this study, we identified a significant relationship of the TyG index with CKD in hypertensive patients with abnormal glucose metabolism, independent of other conventional risk factors. The TyG index in the form of both quartiles and continuous variables was related to CKD. Interestingly, we found the relationship between TyG index and the risk of CKD is non-linear and presents a U-shaped curve. The results were stable and were not modified by gender, age, or BMI, etc. Hence, identification of patients with TyG abnormalities, especially > 8.94, and intervention at an early stage is desirable for prevention of CKD.

First, IR is associated with an increased risk of metabolic diseases, such as hypertension, abnormal glucose metabolism, hyperuricemia, and dyslipidemia (19, 20), which are also risk factors for CKD (21). Second, animal and human studies demonstrated that hyper-insulinemia increases sodium reabsorption, induces glomerular hyperfiltration, which increases renal injury (22–24). Excess can lead to kidney damage. Third, IR can cause and accelerate the progression of CKD by damaging podocytes and basement membrane through chronic inflammation and oxidative stress (25, 26).

Homeostasis model assessment of IR, calculated by insulin and FBG, is often used to assess IR. However, the insulin is not routinely measured in clinical practice, leading that Homeostasis model assessment of IR is inappropriate for clinical practice (13). Therefore, researchers began to study a simple, reliable, and surrogate marker of IR, as TyG.

Our study that baseline TyG index was associated with an increased risk of CKD, which is supported by several studies in general cohorts and patients with DM (27–29), and the results were consistent across genders. However, in a cross-sectional study, only among participants aged over 65 years, higher levels of TyG were correlated with CKD in hypertensive patients (27). However, our study did not find significant difference in the risk of CKD with TyG in different age groups, *P* interaction > 0.05. It may be related to different study designs. Cross-sectional studies could not explain the causal relationship between them, and there were some uncontrolled confounding biases.

Interestingly, our study found a non-linear relationship between TyG index and CKD. With the gradual increase of TyG level, the risk of CKD first fell and after rising until around 8.94 of TyG index and started to increase rapidly afterward. This is similar to the study from China (30). As the TyG quartile increased, the risk of abnormal eGFR also appeared to decrease first and then increase in the study population. On the one hand, as we expected, patients with higher TyG had an increased risk of CKD. On the other hand, our results also suggest that lower TyG levels may increase the risk of CKD. Further studies are needed to elucidate the pathogenesis.

The present study has several strengths. First, we used a longitudinal design to evaluate the association between TyG index and CKD. Second, the study population cohort was at high risk for CKD, and the results may contribute to the prevention and treatment of CKD. Last, this study contributes to expand on reproducible, low-cost, monitoring-tools for the assessment and stratification of patients with a higher CKD risk. However, there were also several limitations in this study: (1) The study was conducted in a single center. However, it was conducted in a regional center for hypertension with patients of a large age range and from all over Xinjiang. (2) The portion of lost to follow-up may potentially bias the results. To minimize the bias, we combined rehospitalization and annual health check-up data to reduce the loss of follow-up rate (17.3% in this study), and there was no significant difference between included subjects and lost follow-up individuals for most baseline information. (3) Although we adjusted for potential risk factors for CKD, other unmeasured or residual confounders, such as genetic susceptibility, may well have affected the findings. However, because the population studied was quite homogeneous, the findings are likely to be stable and reliable. In addition, more attention should be paid to the relationship between cumulative exposure of TyG and CKD in future.

## Conclusion

In conclusion, we found that an elevated TyG index is independently associated with a higher risk of CKD in hypertensive patients with abnormal glucose metabolism. Early intervention of metabolic factors in this high-risk population may prevent the occurrence of CKD, thereby reducing the incidence of CVD and premature death.

## Data availability statement

The raw data supporting the conclusions of this article will be made available by the authors, without undue reservation.

## Ethics statement

Written informed consent was obtained from the individual(s) for the publication of any potentially identifiable images or data included in this article.

## Author contributions

NL and YG contributed to the study concept and design. QZ, YC, LC, JH, and YR contributed to data collection. QZ and YC analyzed the data together and drafted the manuscript. XC, LC, and QL gave important suggestions and did significant changes. All authors reviewed and approved the final manuscript.

## Abbreviations

BMI: Body mass index
BUN: blood urea nitrogen
CI: Confidence interval
CKD: chronic kidney disease
Cr: creatinine
CVD: Cardiovascular disease
DBP: Diastolic blood pressure
DM: diabetes mellitus
FBG: Fasting blood glucose
HbA1c: glycated hemoglobin
HDL-C: High-density lipoprotein cholesterol
HR: Hazard ratio
IR: Insulin resistance
LDL-C: Low-density lipoprotein cholesterol
SBP: Systolic blood pressure
TC: Total cholesterol
TG: Triglyceride
TyG: Triglyceride-glucose
UA: uric acid.
