# Supplementary material for: The non-linear relationship between triglyceride-glucose index and risk of chronic kidney disease in hypertensive patients with abnormal glucose metabolism: A cohort study
Source: Front Med (Lausanne). 2022 Sep 20;9:1018083. doi: 10.3389/fmed.2022.1018083 (PMC9530361; doi:10.3389/fmed.2022.1018083)
Supplement: Supplementary file 1 [file Data_Sheet_1.doc]

Supplement

The nonlinear relationship between triglyceride-glucose index and risk of chronic kidney disease in hypertensive patients with abnormal glucose metabolism: a cohort study

Qing Zhu MD^1,2^, Yuan Chen MD^1,3^ Xintian Cai MD.PHD^1,2^, Li Cai MS^2^, Jing Hong MS^2^, Qin Luo MD^2^, Yingli Ren MS^2^, Yanying Guo MD.PHD^1,3*^, Nanfang Li MD.PHD^2*^[
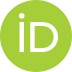
](https://orcid.org/0000-0003-1505-8566)

**Affiliation:**

1. Xinjiang Medical University
2. Hypertension Center of People’s Hospital of Xinjiang Uygur Autonomous Region;

Xinjiang Hypertension Institute;

1. Department of Endocrinology and Metabolic diseases of People’s Hospital of Xinjiang Uygur Autonomous Region

**Corresponding Author**:Nan-fang Li, MD.PHD

Hypertension Center of People’s Hospital of Xinjiang Uygur Autonomous Region;

NO.91 TianChi Road, Urumqi, Xinjiang. 830001

Tel: +86-13999179937; Fax: +86-09918564816

1. mail: [lnanfang2016@sina.com](mailto:lnanfang2016@sina.com) ORCID:https://orcid.org/0000-0003-1505-8566

Yanying Guo MD.PHD

Department of Endocrinology and Metabolic diseases of People’s Hospital of Xinjiang Uygur Autonomous Region

NO.91 TianChi Road, Urumqi, Xinjiang. 830001

E-mail: [guozeyang@126.com](mailto:lnanfang2016@sina.com)

| **Table S1 Baseline characteristics of participants according with and without CKD** | | | | |
| --- | --- | --- | --- | --- |
|  | **Non CKD** | **CKD group** | **Unadjusted HR** | ***P*** |
|  | **n=1731** | **n=302** | **95% CI** |  |
| **Age(years)** | 55.28±11.03 | 56.94±11.11 | 1.02（1.01-1.03） | 0.003 |
| **Male n**（%） | 970(56.0) | 179(59.3) | 0.90（0.72-1.14） | 0.295 |
| **Body mass index(kg/m^2^**) | 27.96±3.91 | 28.59±3.91 | 1.04（1.01-1.07） | 0.007 |
| **Smoking Yes n(%)** | 507（29.3） | 85(28.1) | 0.96（0.74-1.23） | 0.728 |
| **Drinking Yes n(%)** | 466（26.9） | 73(24.2) | 0.89（0.669-1.16） | 0.396 |
| **Druation of hypertension(years)** | 6(2-12) | 9(4-15) | 1.02（1.01-1.04） | 0.001 |
| **Druation of diabetes (years)** | 0(0-2.54) | 0(0-6) | 1.06（1.04-1.08） | ＜0.001 |
| **Systolic BP at bseline (mmHg)** | 147.61±20.99 | 153.66±21.75 | 1.02（1.01-1.02） | ＜0.001 |
| **Diastolic BP at baseline (mmHg)** | 87.51±14.57 | 90.11±15.84 | 1.01（1.00-1.02） | 0.011 |
| **Systolic BP** **follow up (mmHg)** | 142.68±20.78 | 148.43±23.49 | 1.01（1.01-1.02） | ＜0.001 |
| **Diastolic BP follow up(mmHg)** | 84.91±13.86 | 86.34±15.15 | 1.01（0.99-1.02） | 0.078 |
| **Pusle(bits/min)** | 82.01±10.82 | 81.96±11.85 | 1.01（0.99-1.02） | 0.288 |
| **HbA1c**（%） | 6.84±1.24 | 7.35±1.59 | 1.25（1.17-1.33） | ＜0.001 |
| **FBG (mmol/L)** | 6.06±2.11 | 6.86±2.92 | 1.12（1.08-1.16） | ＜0.001 |
| **Cholesterol (mmol/l)** | 4.42±1.09 | 4.52±1.14 | 1.09（0.98-1.20） | 0.114 |
| **Triglyceride(mmol/l)** | 2.11±1.91 | 2.41±2.11 | 1.06（1.03-1.13） | 0.002 |
| **HDL--C(mmol/l)** | 0.98±0.24 | 0.94±0.20 | 0.43（0.25-0.74） | 0.002 |
| **LDL-C(mmol/l)** | 2.62±0.85 | 2.62±0.89 | 0.99（0.86-1.14） | 0.901 |
| **Serum Cr(umol/L)** | 65.19±14.89 | 70.92±18.97 | 1.01（1.00-1.02） | 0.002 |
| **BUN (mmol/L)** | 5.04±1.36 | 5.47±1.56 | 1.18（1.09-1.27） | ＜0.001 |
| **Uric acid (umol/l)** | 330.63±84.88 | 341.57±87.09 | 1.00（1.00-1.00） | 0.004 |
| **Serum K+** （mmol/L） | 3.68±0.28 | 3.66±0.29 | 0.78（0.53-1.14） | 0.205 |
| **TyG** | 8.99±0.67 | 9.21±0.74 | 1.46 (1.25-1.70) | ＜0.001 |
| **Antihypertensive agents n (%)** |  |  |  |  |
| **ACEI/ARB** | 977(56.4) | 198(65.6) | 1.34（1.06-1.70） | 0.015 |
| **CCB** | 1415(81.7) | 262(86.8) | 1.48（1.06-2.06） | 0.022 |
| **Beta-blocker** | 370(21.4) | 73(24.2) | 1.20（0.92-1.56） | 0.176 |
| **Diuretics** | 583(33.7) | 133(44.2) | 1.89（1.51-2.38） | 0.001 |
| **Hypoglycemic therapy n (%)** | 933(53.9) | 205(67.9) | 1.40（1.03-1.92） | ＜0.001 |
| **Lipid-lowering therapy n (%)** | 1412(81.6) | 255(84.4) | 1.83（1.43-2.32） | 0.033 |
| FBG, fasting blood glucose;LDL-C, low-density lipoprotein cholesterol; HDL-C, high-density lipoprotein cholesterol, Cr, creatinine; BUN,Blood urea nitrogen; ACEI, angiotensin-converting -enzyme inhibitors; ARB, angiotensin receptor blockers; CCB, calcium channel blockers | | | | |

| Table S2: Collinearity diagnostics steps | | | | |
| --- | --- | --- | --- | --- |
|  | All indicators | | Not including VIF ＞3 | |
|  | Tolerance | VIF | Tolerance | VIF |
| **Age** | 0.55 | 1.817 | 0.553 | 1.809 |
| **Sex** | 0.435 | 2.296 | 0.442 | 2.264 |
| **BMI** | 0.855 | 1.17 | 0.86 | 1.163 |
| **Smoking** | 0.546 | 1.831 | 0.549 | 1.821 |
| **Drinking** | 0.564 | 1.773 | 0.567 | 1.763 |
| **Druation of hypertension** | 0.74 | 1.351 | 0.741 | 1.349 |
| **Druation of diabetes** | 0.692 | 1.445 | 0.705 | 1.419 |
| **Systolic BP** | 0.49 | 2.042 | 0.492 | 2.031 |
| **Diastolic BP** | 0.416 | 2.404 | 0.417 | 2.400 |
| **Pusle(bits/min)** | 0.907 | 1.103 | 0.908 | 1.101 |
| **FBG** | 0.267 | 3.745 |  |  |
| **HbA1c** | 0.412 | 2.428 | 0.613 | 1.632 |
| **Cholesterol** | 0.123 | 8.102 | 0.649 | 1.541 |
| **Triglyceride** | 0.158 | 6.314 |  |  |
| **HDL-C** | 0.647 | 1.545 | 0.823 | 1.215 |
| **LDL-C** | 0.168 | 5.952 | 0.897 | 1.115 |
| **Serum Cr** | 0.519 | 1.926 | 0.524 | 1.908 |
| **BUN** | 0.763 | 1.311 | 0.767 | 1.304 |
| **Uric acid** | 0.695 | 1.44 | 0.707 | 1.415 |
| **Serum potassium** | 0.916 | 1.092 | 0.916 | 1.091 |
| **ACEI/ARB** | 0.884 | 1.131 | 0.886 | 1.128 |
| **CCB** | 0.904 | 1.107 | 0.904 | 1.106 |
| **Beta-blocker** | 0.916 | 1.092 | 0.918 | 1.089 |
| **Diuretics** | 0.877 | 1.14 | 0.878 | 1.139 |
| **Hypoglycemic therapy** | 0.697 | 1.434 | 0.711 | 1.407 |
| **Lipid-lowering therapy** | 0.93 | 1.075 | 0.932 | 1.073 |
| TyG | 0.171 | 5.84 | 0.698 | 1.433 |
| FBG, fasting blood glucose;LDL-C, low-density lipoprotein cholesterol; HDL-C, high-density lipoprotein cholesterol, Cr, creatinine; BUN,Blood urea nitrogen; ACEI,angiotensin-converting-enzyme inhibitors; ARB, angiotensin receptor blockers; CCB, calcium channel blockers | | | | |
